# Dogs Do Not Show Pro-social Preferences towards Humans

**DOI:** 10.3389/fpsyg.2016.01416

**Published:** 2016-10-04

**Authors:** Mylène Quervel-Chaumette, Gaëlle Mainix, Friederike Range, Sarah Marshall-Pescini

**Affiliations:** Comparative Cognition, Messerli-Research Institute, University of Veterinary Medicine of ViennaAustria

**Keywords:** pro-social behaviors, cooperation, dogs, interspecific context, human partners

## Abstract

Pro-social behaviors are defined as voluntary actions that benefit others. Comparative studies have mostly focused on investigating the presence of pro-sociality across species in an intraspecific context. Taken together, results on both primates and non-primate species indicate that reliance on cooperation may be at work in the selection and maintenance of pro-social sentiments. Dogs appear to be the ideal model when investigating a species’ propensity for pro-sociality in an interspecific context because it has been suggested that as a consequence of domestication, they evolved an underlying temperament encouraging greater propensity to cooperate with human partners. In a recent study, using a food delivery paradigm, dogs were shown to preferentially express pro-social choices toward familiar compared to unfamiliar conspecifics. Using the same set-up and methods in the current study, we investigated dogs’ pro-social preferences toward familiar and unfamiliar human partners. We found that dogs’ pro-social tendencies did not extend to humans and the identity of the human partners did not influence the rate of food delivery. Interestingly, dogs tested with their human partners spent more time gazing at humans, and did so for longer after food consumption had ended than dogs tested with conspecific partners in the initial study. To allow comparability between results from dogs tested with a conspecific and a human partner, the latter were asked not to communicate with dogs in any way. However, this lack of communication from the human may have been aversive to dogs, leading them to cease performing the task earlier compared to the dogs paired with familiar conspecifics in the prior study. This is in line with previous findings suggesting that human communication in such contexts highly affects dogs’ responses. Consequently, we encourage further studies to examine dogs’ pro-social behavior toward humans taking into consideration their potential responses both with and without human communication.

## Introduction

Helping one another occurs routinely in humans and can be directed toward complete strangers (Fehr and Fischbacher, 2003; Silk and House, 2011). Recently, researchers have started to investigate the evolutionary origin of pro-sociality, defined as voluntary actions that benefit others, by studying mainly non-human primate species. However, controversial results render conclusions about both proximate and ultimate mechanisms difficult to draw (Silk and House, 2011; Cronin, 2012; Marshall-Pescini et al., 2016 for a review). Nonetheless, dependence on a species reliance on cooperative activities (e.g., cooperative breeding and alloparental care) appears to be an important factor in the occurrence and maintenance of pro-social behaviors at least in primate species (Burkart et al., 2014), suggesting that other, non-primate species, that rely on cooperation might also show a higher propensity toward pro-sociality.

In this respect, canids may represent a good model with which to investigate pro-sociality due to their high level of sociability and the presence of allo-maternal care, joint territory defense, and group hunting in a number of species (Creel et al., 1997; Bonanni et al., 2010; MacNulty et al., 2014; Paul et al., 2014). Indeed, using a pro-social choice task commonly adopted in primate studies, “the bar-pulling paradigm,” we recently demonstrated that pet dogs exhibit other-regarding preferences toward familiar conspecific partners (Quervel-Chaumette et al., 2015). In this original study, dogs sitting in a donor enclosure were first trained to pull on a baited tray and then retrieve the piece of food from an adjacent receiver enclosure. During the testing phase, however, donor dogs could no longer access the receiver enclosure, which was instead occupied by either a familiar or a stranger dog. We could have shown that pet dogs deliver more food to the receiver enclosure when a familiar compared to a stranger conspecific could access the food. Importantly, we included stringent control conditions to exclude the possibility that desire for social contact or social facilitation effects drove subjects’ pro-social behaviors thus also excluding the possibility that the increased pro-social behavior was just a byproduct of other behaviors. In addition, knowledge-probe trials were run to ensure that dogs understood the contingencies of the task.

Since dogs also share their ecological niche with humans, they represent a good model also to investigate pro-sociality in an interspecific context. In fact, it has been suggested that, as a consequence of domestication, dogs evolved special socio-cognitive skills enabling them to communicate and cooperate with humans (Hare et al., 2002; Hare and Tomasello, 2005). Indeed with adequate socialization and specific training, dogs can show behaviors that are functionally helpful to humans such as guiding blind people, rescuing people or supporting people during hunting. Furthermore, in an experimental set-up Bräuer et al. (2013) showed that pet dogs would help a human to retrieve an object by pressing a button. However, this behavior occurred only when the human gave strong communicative cues by pointing at the button itself, suggesting that dogs may have perceived the situation as an ‘obedience’ task. In a follow up study, the authors conducted a condition in which humans could express their needs in a more naturalistic manner but without directly pointing at the button. In this situation, the helping behavior of the dogs was much higher, suggesting that expressing interest in the “reward” may, to some extent, elicit dogs’ pro-social behaviors. However, it makes conclusions about the pro-social motivation of the dogs toward the human partner difficult to draw, because it is still unclear whether dogs’ activity levels were simply elicited by a higher arousal, when encouraged by the human to act, only incidentally resulting in a more pro-social behavior. Removing human communication from the experimental context and hence the risk that dogs are simply ‘obeying’ the human’s request or becoming more active due to increased arousal may help to clarify dogs’ pro-social attitudes toward humans.

Consequently, in the current study, we investigated the pro-social preferences of dogs toward humans when no social communication was involved. Considering our previous study using the bar-pulling paradigm showing that dogs behave pro-socially toward conspecifics that showed little communication toward them, we here used the exact same methods and set-up in order to test dogs’ prosocial behavior toward human partners. Moreover, because there is considerable evidence that dogs develop a preferential relationship with their owners (Parthasarathy and Crowell-Davis, 2006; Palmer and Custance, 2008; Konok et al., 2011; Horn et al., 2013) and given the fact that dogs in our previous study were more pro-social toward their familiar conspecific partner (owner) than a stranger, here we tested another group of dogs both with their owners and with an unfamiliar human, thereby assessing if familiarity also influences dogs’ pro-social choices toward humans. Because the dogs showed prosocial behavior toward familiar conspecifics and demonstrated understanding of the contingencies of the task, we expected them to show similar prosocial behavior toward a familiar human if they were inclined to do so.

Finally, because the identical methods were adopted both in the current study and in the study assessing pro-sociality toward a familiar and stranger conspecific, we compared results to investigate whether dogs would show similar pro-social concerns toward both conspecific and human partners. This comparison may help to further our understanding of both the evolutionary origin of such behavior in dogs, and dogs’ appraisal of human vs. conspecific partners when placed in a cooperative context involving food.

## Materials and Methods

### Ethical Approval

All procedures were approved by the institutional ethics committee in accordance with GSP guidelines and national legislation (Ref. 09/01/97/2014).

### Subjects

All testing took place at the Clever Dog Lab of the Messerli Research Institute at the University of Veterinary Medicine Vienna, Vienna, Austria. 18 dogs (nine females and nine males) of various ages and breeds participated in the study (Supplementary Table S1). All dogs were pets and lived in a household with their owner. Seven of the 18 dogs shared the household with another dog. All subjects were initially naïve to the study and set-up.

### General Procedure and Set-up

**FIGURE 1:**
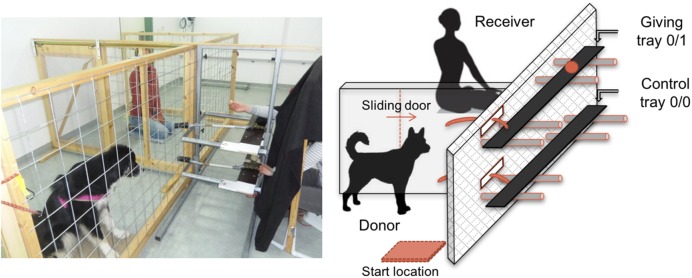
**Side view of the experimental set-up**.

Dogs were placed in one of two side-by-side enclosures, while the human (owner or stranger) was placed in the other enclosure. The enclosures, which were separated by wire mesh and a Plexiglas door, allowed dogs to clearly see and smell the human partner when s/he was present in the receiver enclosure (**Figure 1**). A bar-pulling device consisting of two movable shelves (one above the other) was placed in front of the two enclosures. The experimenter (E) sitting behind the bar-pulling device was made invisible by a black curtain to prevent them from influencing the donor-dog’s choice. Before each trial, the experimenter, whilst remaining invisible, gave a command to the dogs to go and sit on a “start location” from where they could clearly see the baiting process. Through holes in the curtain, E could place a piece of food on one of the two shelves in front of the receiver enclosure. Then the two ropes connected to the platforms were simultaneously released in the donor enclosure and the dogs had 5 s to make a choice. Pulling the giving-tray (0/1) delivered the food to the receiver enclosure. Alternatively, not pulling within the allotted time or pulling the empty tray (0/0) did not deliver food to the partner. In the latter cases, the experimenter removed the food and proceeded to the next trial. For the entire experiment, the food used was a piece of cheese.

### Training Phase

During the training phase, the door between the two adjacent enclosures was open. Donor-dogs were trained to observe the baiting process from the start location, pull on the baited tray (giving) and retrieve the food reward by moving into the receiver enclosure. Dogs had to successfully pull the baited tray and retrieve the reward in the receiver enclosure in 17 out of 20 trials in order to proceed to the testing phase.

### Testing Phase

The testing phase consisted of five motivation sessions interspersed with the five experimental conditions. Both motivation sessions and experimental conditions were conducted on different days.

#### Motivation Sessions

Because our experiment was based on an extinction-like task, we conducted a motivation session prior to each experimental condition in order to re-establish the donor-dogs’ motivation and performance. These motivation sessions mirrored the training phase in that the donor had access to both enclosures and hence could access the food in the receiver enclosure if the giving-tray was selected. Motivation sessions comprised 20 trials where dogs had to pull the baited tray at least 17 times (Probability of success = 0.85, *p* < 0.001) before they were allowed to proceed to the next experimental condition.

#### Experimental Conditions

In these conditions, the door in between the two adjacent enclosures was closed. Consequently, donor-dogs could not access the food in the receiver enclosure after pulling the giving-tray.

**FIGURE 2:**
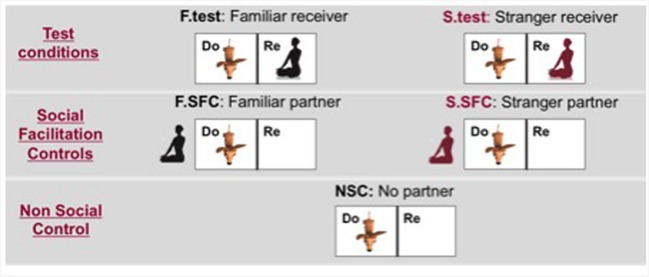
**Location of the donor-dog and human partners in each condition (Do, Donor enclosure; Re, Receiver enclosure; *F*.test, familiar test condition; F.SFC, Social Facilitation Control with the familiar human; *S*.test, Stranger test condition; S.SFC, Social Facilitation Control with the familiar human; NSC, Non Social Control)**.

On separate days, each donor-dogs carried out five experimental conditions (see **Figure 2**):

1. In the *familiar test condition*, the owner sat in the receiver enclosure. The owner was instructed to sit on a carpet on the floor in the middle of the receiver enclosure facing the bar-pulling apparatus with the palms of their hands in their laps and facing upward. Any communication, including eye contact, from the owner toward the dog was forbidden. Importantly, when the giving-tray was pulled by the donor-dog, the owner was instructed to take the food and eat it. While eating, the owner was forbidden from producing any vocal sounds only the natural noises from the chewing were permitted. Despite being discouraged to actively communicate with the dogs, all humans happily ate the food, not showing any signs of disgust. After consumption of the treat, the owner was instructed to place their hands in the original position.
2. In the *stranger test condition*, a human unknown to the donor-dogs but matching the gender of the owner was present in the receiver enclosure instead of the owner. The strangers were instructed to behave in the same way described above. During these tests the owner was outside the testing room.
3. In the *social facilitation control with the familiar human*, the owner sat one meter away on the other side of the donor enclosure. This condition allowed us to establish whether the mere presence of a partner enhances dogs’ pulling behavior. As in test conditions owners were required to sit with their hands in their lap and show no communicative behaviors toward their dog.
4. In the *social facilitation control with the stranger partner*, the procedure was exactly the same as described above, except that the unknown human replaced the owner. During this condition, the owner stayed outside the testing room.
5. In the *non-social control*, the receiver enclosure was empty and no human partners (except the experimenter behind the curtain) were present in the experimental room.

Each experimental condition comprised a maximum of 40 trials. A session ended if for five consecutive trials donor-dogs did not pull on either tray or if they refused to sit back at the start location prior to the next trial, despite five consecutive requests from the experimenter. When the session ended, donor-dogs and their partners (when present) remained in their original locations and four “knowledge-probe” trials were run. In these four trials, the food was placed on the side of the tray in front of the donor-dog enclosure. Hence, pulling the baited tray would deliver food to the donor-dog enclosure. In those trials, we expect dogs to behave in a way consistent with past reinforcement contingencies, in this case pulling the baited tray. If they do so, it additionally insures that donor-dogs are comfortable to pull the rope to deliver food to themselves in the presence of both a familiar and stranger human partners.

Each donor-dog participated in one session per condition, presented in a semi-randomized and counterbalanced order. Indeed, the two conditions involving a specific partner (test condition and social facilitation control) were run one after the other such that donors starting with the test condition next received the related social facilitation control and vice versa (e.g., familiar test followed by familiar social facilitation control or vice versa). The non-social control was randomly run before, between or after the two conditions involving a human partner. Importantly, we counterbalanced which conditions (familiar/stranger) were run first across subjects.

### The Initial Dog–Dog Pro-social Study

In the initial study, the exact same material and methods were used, except that the group of dogs tested was paired with familiar and stranger conspecifics. We tested 16 donor dogs of various ages and breeds. The familiar conspecific was a dog living with the subject for at least 1 year before they were tested (Supplementary Table S2 for further information). We used little pieces of sausage as food rewards.

#### Statistical and Behavioral Analyses

For each condition, we looked at three dependent variables: (i) the number of trials, (ii) the number of total pulls (including the pulling of both the empty tray and the giving-tray), and (iii) the number of giving-pulls.

A Pearson correlation revealed that the three variables were highly correlated (Supplementary Table S3). Because the number of giving-pulls represents the actual food donation across conditions, it is the best measure of a dog’s pro-social choices. This variable was, therefore, chosen as the dependent measure in the following analyses.

We first tested whether the type of condition run and the number of sessions influenced the number of giving-pulls performed by the dogs. We hence ran generalized linear models (LMM) with the identity of each dog as a random factor and the number of giving-pulls as our response variable. We controlled for over-dispersion by using the glmmADMB package and function. As explanatory variables we used, the order of sessions, the type of condition and the interaction between session and condition. We selected models of best fit by using a likelihood ratio test (R function “ANOVA”).

The video recordings of the experimental conditions were coded with Solomon Coder Beta 15.01.13 (Copyright András Péter^1^). We coded all occurrences of scratching, yawning, lip-licking, and attempting to leave the donor enclosure, and combined them in a single category dubbed ‘stress behaviors.’ The duration of dog’s vocalizations (whines and barks), as well as the time each dog spent gazing at the human (familiar and stranger) in the conditions involving a partner, were also coded. Inter-observer reliability was tested on twenty percent of the videos by a second experimenter (all Cohen Kappa were above 0.88).

For the behavioral variable, General LMM using the function lmerTest of the R package lme4 with the donor’s identity as a random factor were run. We used the following response factor terms: the mean duration of gazing toward the partner, the mean duration of vocalization and the average frequency of stress behavior (boxcox transformation, λ = -1,47). The order of session, the type of condition run as well as the interaction between session and condition were included in the full model as explanatory variables. We selected models of best fits by using the “ANOVA” function (*F*-values and *p*-value reported in the results).

## Results

### Number of Giving-Pulls

**FIGURE 3:**
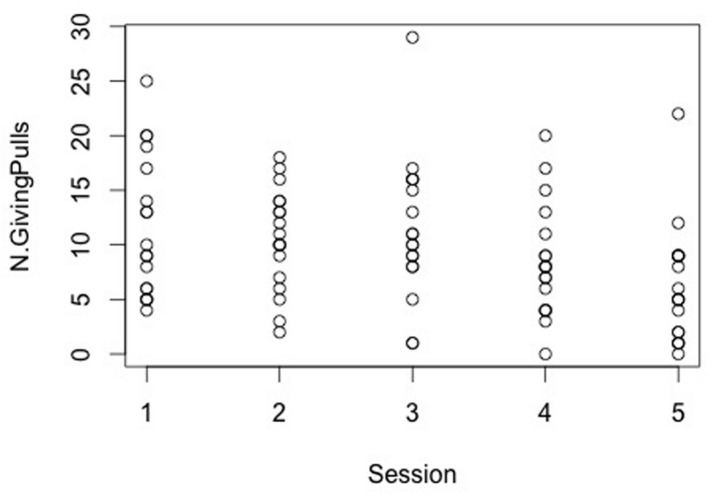
**The number of giving pulls decreases across session (likelihood ratio test: χ^2^ = 21.71; *p* < 0.001)**.

The model revealed no session by condition interaction effect on the response variable (**Table 1**). However, a session effect emerged with results showing that the pulling of the giving-tray decreased from the first to the last session (glmm: *z* = -3.73, *SE* = 0.039, *p* < 0.001, **Figure 3**), whereas the condition did not affect the number of giving-pulls performed by dogs (**Table 1**).

**Table 1:** Likelihood ratio test of the GLMM models and ANOVA output of the LMM models.

	Main factors		
	Session-Condition interaction	Session	Condition
Explanatory variables	GLMM		
Number of giving pulls	Likelihood ratio test: χ^2^= 4.03 *p* = 0.5	Likelihood ratio test: χ^2^= 21.71 *p* < 0.001^∗^	Likelihood ratio test: χ^2^= 3.18 *p* = 0.53
	LMM		
Vocalizations	*F*_ (4,71)_ = 0.09 *p* = 0.98	*F*_(1,67)_ = 0.20 *p* = 1.65	*F*_(4,67)_ = 1.54 *p* = 0.20
Stress behavior	*F*_(4,74)_ = 1.01 *p* = 0.41	*F*_(1,71)_ = 0.15 *p* = 0.69	*F*_(4,68)_ = 4,23 *p* = 0.58
Gazing time toward the human	*F*_(3,64)_ = 1.08 *p* = 0.36	*F*_(3,67)_ = 0.02 *P* = 0.88	*F*_(3,67)_ = 27.59 *p* < 0.001^∗^

### Performance of Dogs on the Knowledge Probe Trials

In the four knowledge-probe trials at the end of each session, all subjects always pulled the baited tray delivering food into their own enclosure showing that dogs were still motivated to pull if they could access the food themselves.

### Behaviors

For all the response variables tested, no interaction between the order of sessions and condition were found (**Table 1**). The frequency of stress behavior as well as the duration of dogs’ vocalizations was not influenced by the order of sessions or the condition (**Table 1**).

However, condition affected the time dogs spent gazing toward the human (**Table 1**). Indeed, dogs spent more time looking at the human during the test conditions compared with the social facilitation controls (LMM_(strangerpartner)_: β = -28.77, *SE* = 4.4, *df* = 68, *p* < 0.001; LMM_(familiarpartner)_: β = -28.55, *SE* = 4.4, *df* = 68, *p* < 0.001), i.e., when the humans were present but outside of the receiver enclosure and, consequently, unable to access the food when the giving-tray was pulled. There was, however, no effect of the identity of the human on gazing behavior of the dogs (LMM: β = -1.11, *SE* = 4.4, *df* = 68, *p* = 0.802).

### Between Group Comparison: Do Dogs Give More to Human or Conspecific Partners?

**FIGURE 4:**
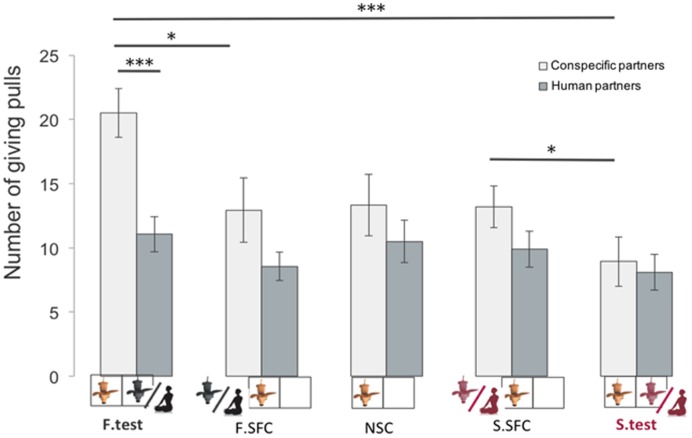
**Mean number of giving-pulls (mean ± SEM, *n* = 16 individuals for the experiment 1 and *n* = 18 for the second experiment) in which donors pulled the giving (0/1) tray across sessions (^∗^*p* < 0.05, ^∗∗^*p* < 0.01, ^∗∗∗^*p* < 0.001)**.

**FIGURE 5:**
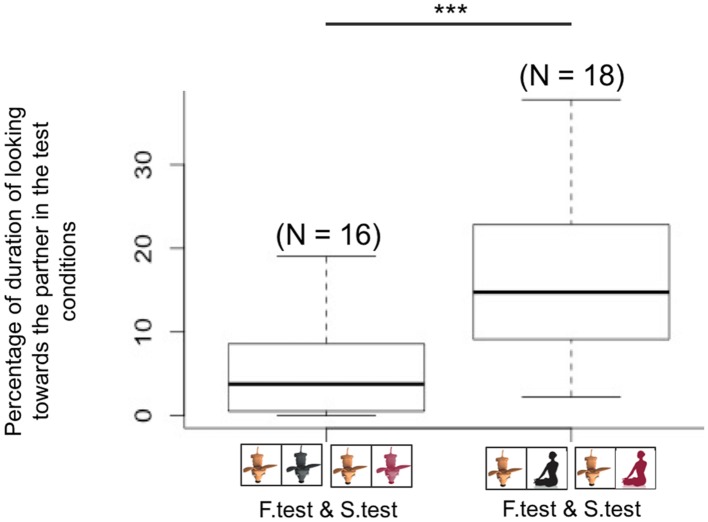
**Dogs paired with conspecific partners spent less time looking at their partner than dogs paired with humans in both test conditions (familiar and stranger test conditions, ^∗∗∗^*p* < 0.001)**.

When directly comparing the giving behavior of the group of dogs paired with conspecifics with the group paired with humans, we found a difference only in the familiar test condition. Indeed, dogs paired with familiar conspecifics delivered more food (mean ± SE = 20.5 ± 1.9) than dogs paired with the owner (mean ± SE = -11.3 ± 1.4; glmm: *z* = 3.72; *p* < 0.001, **Figure 4**). In all other conditions there were no differences in the rate of pulling for conspecifics compared to humans. To better understand why dogs did not preferentially provide food to their familiar human partners, we looked at potential behavioral differences between dogs tested with humans and conspecifics in the test conditions, where the partner (either familiar or stranger) was present in the receiver enclosure. Results showed that dogs paired with human partners spent more time gazing at their partners than dogs paired with conspecifics (l mm: β = 7.838, *SE* = 2.469, *df* = 29, *p* = 0.003, see **Figure 5**), suggesting that dogs perceived the two partners (conspecifics vs. humans) differently. It could be argued that dogs spent more time looking at the human partners compared to conspecific ones simply because humans may have needed a longer time to manipulate and eat the food. Hence we additionally coded the average time owners and familiar conspecifics spent eating one piece of food per trials as well as the percentage of feeding per session, and ran further analyses to examine whether this influenced the time dogs spent looking at their partners. Results indicated that the average time owners and familiar partners needed to eat one piece of food was not significantly different (human: from when the hand touched the food until the end of the chewing: mean ± SE = 4.5 ± 0.30 s per trials; familiar conspecifics: when the mouth reached the platform until the end of the chewing or licking the tray: mean ± SE = 3.6 ± 0.18 s per trials, Wilcoxon test: *W* = 56.5, *p* = 0.07). Moreover, the percentage of time dogs spent looking at the owner per session (mean ± SE = 17.92 ± 2.28) was significantly higher than the percentage of time owners needed to eat the food when delivered (mean ± SE = 12.84 ± 1.23, pairwise Wilcoxon: *W* = 28, *p* = 0.01). Conversely, the percentage of time dogs spent looking at the familiar conspecific (mean ± SE = 6.81 ± 1.28) was not significantly different from the percentage of time the familiar conspecifics spent eating the food during the test (mean ± SE = 6.97 ± 0.54, pairwise Wilcoxon: *W* = 58, *p* = 0.63).

## Discussion

Using the same pro-social choice paradigm as the one used here, our previous study demonstrated that pet dogs preferentially demonstrate other-regarding preferences toward familiar conspecifics. Here, we found that dogs did not extend their pro-social behavior toward human partners. Furthermore, the identity of the human partner did not influence the rate of food delivery performed by the dogs. Importantly, we found that dogs spent more time gazing at humans than at conspecific partners, which may explain why dogs stopped pulling the giving-tray sooner for their owners than for familiar conspecifics.

The observed lack of pro-social behavior toward human partners may be explained by the difficulties dogs might have had in understanding the task. It is possible that dogs did not distinguish between the different situations and simply relied on the fact that, regardless of the condition, pulling the giving tray was no longer rewarded. Indeed, despite the fact that dogs regained their initial level of motivation before entering each control/test session, the only effect we observed was a decrease in the number of giving-pulls across sessions independent of condition.

However, two lines of evidence suggest that dogs did understand the contingencies of the task and perceived the consequence of their actions. Firstly, at the end of each session, regardless of condition, all dogs always pulled in “knowledge probe” trials when food was delivered to themselves. The consistency of these results shows that dogs could distinguish the consequence of their action in relation to where the food was placed on the apparatus, and they quickly regained motivation to pull the tray when this benefitted them (rather than the partner). Second, when the human partner was sitting in the receiver enclosure (test conditions), dogs spent more time looking at the human than in the social facilitation controls, when the human was present but could not access the food. This indicates that dogs were attentive to where the food was delivered and closely observed the consequence of their pulling actions namely that the human was happily eating the food in the test conditions.

However, it could be argued that the looking behavior may simply be the result of a sign-tracking effect defined as individuals being more likely to stare longer at places where they have previously received food (Flagel, 2015). However, dogs paired with conspecifics in the initial study spent less time gazing at their partner located in the receiver enclosure (Quervel-Chaumette et al., 2015) than the dogs paired with human receivers in the current experiment. Consequently, it is unlikely that dogs’ staring at the human in the test situation was a simple by-product of a sign-tracking effect.

Alternatively, it could be argued that the greater duration of looking behavior toward humans compared to conspecifics might have been driven by the fact that humans needed more time to eat the food reward than did the conspecific partners. However, further analysis revealed that this was not the case. Results additionally indicate that, whereas dogs stopped looking at the conspecific partner when it stopped eating, looking toward the human partner continued even after s/he had finished the food.

Accordingly, the looking behavior is likely not a by-product of some other action, but rather indicates that the dogs were looking at the humans for information. This could simply be due to expectation of being rewarded for their actions. The dogs likely have a history of positive reinforcement for any positive interaction with humans, which may include looking at them.

Moreover, in human-dog interactions, dogs’ gazing at humans is usually interpreted as a “search for help” or information-seeking behavior (Udell, 2015). We indeed know that dogs are highly sensitive and attentive to human communicative cues (Udell and Wynne, 2008). Furthermore, a number of studies have also shown that dog’s activity levels (Range et al., 2009), for example in terms of manipulating an apparatus (Bräuer et al., 2013; Udell, 2015) are higher when the human uses vocal and bodily cues to encourage them. Indeed dogs in the Bräuer et al. (2013) study were shown to activate a door allowing the person to retrieve a desired object only, when the partner was communicating with them in a naturalistic way. In the current study, the human was instructed to refrain from praising, talking to or even glancing at the dog. It is possible that the lack of communication led to the extinction of the pulling behavior observed since behaving in a pro-social manner did not appear to act as a reinforcer for the dog.

Consequently, the pulling behavior of the dogs when paired with humans was potentially first motivated by the expectation of obtaining the food from the human, but was extinguished when dogs were repeatedly confronted with the human ignoring them potentially, because the lack of communication may have been perceived as a negative feedback by the dogs. Dogs paired with familiar conspecifics, may also have initially expected to obtain food for themselves (given they also had previous experience in motivation trials of obtaining food for themselves). However, differently from dogs tested with humans, dogs tested with their familiar conspecifics, once they observed their partner eating the food, nevertheless continued delivering food to them for longer than in the control conditions. The inclination of dogs to deliver food to their familiar conspecific partners may be partly driven by prior successful food sharing experiences. Indeed, it is possible that animals that are routinely fed at the same time or regularly feed together may have a positive association with such experiences which increases their propensity to express pro-social behavior in such tasks.

Besides, humans partner did not express any desire or interest toward the food. Some authors have argued that the signaling of needs helped the actor to understand the others goal and consequently act in a pro-social manner (Pelé et al., 2009; Schwab et al., 2012; Bräuer et al., 2013). However, other studies found that reaching for food or requests did not, or even prevent, the expression of pro-social acts (Silk et al., 2005; Burkart et al., 2007; Vonk et al., 2008; Cronin et al., 2009). Similarly in our previous study, dogs’ receiver reaching for food did not increase the donors’ rate of giving (Quervel-Chaumette et al., 2015) suggesting that this was not a requirement for dogs to understand the task and hence show pro-social behavior. Therefore, even though the current set-up does not allow us to strongly confirm that dogs identified the human’s interest into food, we do not think that this explains their lack of pro-sociality, especially since they witness the human eating the piece of cheese following their pulling action. Accordingly, the only association they could have made between the human and the food is that he/she would be willing to eat the food when becoming available. Moreover, on a daily basis dogs observe regularly that humans eat food and often cheese making it highly unlikely that the dogs assumed that humans did not want the food during the test.

Importantly, whether sausage (in the conspecific study) or cheese was used, both rewards are highly valuable for dogs, and both are used regularly by owners to reward their dogs. Additionally, in both studies dogs were trained with the same food that was later used during the test, and thus have equal experience with the food in both studies. Later, during the test, both group of dogs observed the partner eating that food. Accordingly, this is unlikely that dogs did not realize that the humans were eating the food and that they like cheese (same as the conspecifics with the piece of sausage), and nonetheless they decided not to give additional food to their owners. So, in the current study, dogs were not pro-social toward humans even though they had the same information as the dogs in the previous study with the conspecifics.

However, it is possible that the similarity in behavior and expressions between conspecifics in contrast to a human partner following the feeding process was higher. It is hence possible that the donor dog perceived the familiar conspecific’s excitement and experienced a form of emotional contagion encouraging the next pro-social behavior in the following trials. Since emotional contagion has been shown to be more likely to occur in contact of familiar partners this would additionally explain why dogs’ pro-social preferences were biased in favor of familiar conspecifics in our initial study. If indeed similarity in body features and expressions is needed to experience this form of emotional contagion phenomenon, it might explain why dogs did not react pro-socially with humans but with conspecifics. To further examine this hypothesis, future studies should include the collection of physiological measurement. So far, this remains a hypothesis and more research needs to be done in order to identify whether emotional contagion is indeed an underlying mechanism of pro-sociality in dogs.

## Conclusion

The commonly adopted pro-social choice paradigm, we show that the pro-social concerns of dogs are largely limited to familiar conspecifics. Although selection of dogs during the process of domestication shaped their temperament profile to form close social bonds with humans, when food is involved and the human is silent, dogs fail to behave pro-socially toward them. We know from other studies that the human communication in such contexts (Bräuer et al., 2013), as well as the type of experimental set-up adopted when investigating pro-social behavior, can highly affect donor responses (Cronin, 2012; Bräuer et al., 2013; and Marshall-Pescini et al., 2016 for review). Consequently, we encourage further studies to examine pro-social behavior of dogs toward humans in potentially more naturalistic setting, which does not involve feeding.

## Author Contributions

MQ-C, FR, SM-P designed the experiment. MQ-C performed the statistical analyses, wrote the paper, coded all the videos and drew the figure included in the article. MQ-C and GM conducted the experiment. GM coded 20 percent of the videos. FR and SM-P helped writing the paper.

## Conflict of Interest Statement

The authors declare that the research was conducted in the absence of any commercial or financial relationships that could be construed as a potential conflict of interest.

## Footnotes

1 http://solomoncoder.com
