# Supplementary material for: Dogs Do Not Show Pro-social Preferences towards Humans
Source: Front Psychol. 2016 Oct 4;7:1416. doi: 10.3389/fpsyg.2016.01416 (PMC5047953; doi:10.3389/fpsyg.2016.01416)
Supplement: Supplementary file 1 [file Data_Sheet_1.DOCX]

SUPPLEMENTARY MATERIAL

Dogs do not show pro-social preferences towards humans.

Mylène Quervel-Chaumette^1*^, GaëlleMainix, Friederike Range^1^, Sarah Marshall-Pescini^1^Affiliations

^1^Comparative Cognition, Messerli Research Institute, University of Veterinary Medicine, University of Vienna, Vienna, Austria. 1 Veterinärplatz, 1210 Wien (Austria).

*Correspondence: MylèneQuervel-Chaumette, [mylene.chaumette@vetmeduni.ac.at](mailto:mylene.chaumette@vetmeduni.ac.at)

Table.S1. Dog-Human study: Age, sex and breed of the donor’s dogs participating in the current experiment

| **Donor’s dogs** | **Donor’s Sex** | **Donor’s Age** | **Donor’s breed** |
| --- | --- | --- | --- |
| Monty | Male | 6 | Border Collie |
| Cameron | Male | 5 | Border Collie |
| Kilio | Male | 6 | Mix breed |
| Benji | Male | 6 | Mix breed |
| Knight | Male | 4 | Australian Shepherd |
| Boby | Male | 9 | Boxer |
| Ted | Male | 2 | Border Collie |
| Gatsby | Male | 4 | Border Collie |
| Aiko | Male | 5 | Australian Shepherd |
| Lola | Female | 4 | Border Collie mix |
| Amy | Female | 7 | Border Collie |
| Muffin | Female | 5 | Mix breed |
| Akina | Female | 8 | Akita |
| Asta | Female | 8 | EntlebucherSennenhund |
| Helena | Female | 5 | Poodle |
| Chilli | Female | 6 | Australian Shepherd |
| Tiara | Female | 3 | Border Collie |
| Miley | Female | 6 | Border Collie |

Table.S2. Dog-Dog study: Age, sex and breed of the donor’s dogs participating in the initial experiment

| **Donor's dogs** | **Donor’s Sex** | **Donor’s Age** | **Donor’s breed** |
| --- | --- | --- | --- |
| Finn | Male | 11 | Australian Shepherd |
| Luke | Male | 10 | Border Collie |
| Joker | Male | 8 | Border Collie |
| Ultimo | Male | 6 | Border Collie |
| Talie | Male | 4 | Husky |
| Casper | Male | 3 | Border Collie |
| Mago | Male | 11 | Golden Retriever |
| Bounty | Female | 9 | Australian Shepherd |
| Achuck | Female | 8 | Chesapeake bay retriever |
| Chio | Female | 8 | Border collie |
| Neela | Female | 7 | Australian Shepherd |
| Chasie | Female | 6 | Border Collie |
| Faye | Female | 6 | Border Collie |
| Mali | Female | 6 | Mixed breed |
| Sunny | Female | 3 | Mixed breed |
| Flappi | Female | 6 | Pumi-Mixed breed |

Table S3. Matrix of Pearson’s correlation coefficient between the three potential dependant variables.

| **Pearson’s correlation coefficient** | **Number of trials** | **Number of total pulls** | **Number of giving pulls** |
| --- | --- | --- | --- |
| Number of trials | NA | NA | NA |
| Number of total pulls | R=0.93 | NA | NA |
| Number of giving pulls | R=0.92 | R=0.99 | NA |
